# Structural Basis for Substrate-specific Acetylation of Nα-acetyltransferase Ard1 from *Sulfolobus solfataricus*

**DOI:** 10.1038/srep08673

**Published:** 2015-03-02

**Authors:** Yu-Yung Chang, Chun-Hua Hsu

**Affiliations:** 1 Department of Agricultural Chemistry, National Taiwan University, Taipei 10617, Taiwan; 2 Center for Systems Biology; Genome and Systems Biology Degree Program, National Taiwan University, Taipei 10617, Taiwan

## Abstract

Nα-acetyltransferases (Nats) possess a wide range of important biological functions. Their structures can vary according to the first two residues of their substrate. However, the mechanisms of substrate recognition and catalysis of Nats are elusive. Here, we present two structure of *Sulfolobus solfataricus* Ard1 (SsArd1), a member of the NatA family, at 2.13 and 1.84 Å. Both structures contain coenzyme A, while the latter also contains a substrate-derived peptide. Sequential structure-based mutagenesis revealed that mutations of critical residues for CoA binding decreased the binding affinity of SsArd1 by 3 ~ 7-fold. Superimposition of SsArd1 (NatA) with human Naa50p (NatE) showed significant differences in key residues of enzymes near the first amino-acid position of the substrate peptide (Glu35 for SsArd1 and Val29 for Naa50p). Further enzyme activity assays revealed that the substrate specificity of SsArd1 could be altered from SSGTPT to MEEKVG by a range of Glu35 mutants. These studies provide not only a molecular elucidation of substrate recognition and specificity for the NatA family, but also insight into how members of the NAT family distinguish between amino acids at the substrate N-terminus from the ancient monomeric archaeal Ard1.

The co-translational modification of N-terminal acetylation is ubiquitous in all kingdoms and has critical biological functions, including as a signal for protein degradation, protein-protein interaction, protein translocation, and apoptosis^1^,^2^,^3^,^4^,^5^,^6^,^7^,^8^. Nα-acetyltransferase (Nat) performs this modification and transfers an acetyl group from acetyl-coenzyme A (acetyl-CoA) to an α-amino group from the first residue of the protein substrate. In eukaryotic cells, Nat complexes are composed of one catalytic and one or more auxiliary subunits, and they are involved in protein Nα-acetylation. The 6 Nat complexes (NatA-F) are conserved from yeast to mammalian cells and differ on the basis of substrate specificity^9^,^10^,^11^. However, the detailed mechanism of substrate recognition and catalysis for Nat complexes is unclear.

In yeast, NatA complex, consisting of Ard1 and Nat1^12^, may acetylate many but not all proteins, beginning with small residues such as Ala, Ser, or Thr^12^,^13^,^14^. The mammalian NatA complex is composed of the catalytic subunit Naa10p and auxiliary subunit Naa15p and prefers to acetylate the N-terminal end with a Ser substrate^15^,^16^. However, the monomeric Naa10p in an uncomplexed form acetylates the N-terminal end with an acidic residue instead of the classical Ser-type substrates^15^. Comparison of the crystal structure of the complexed and uncomplexed forms of catalytic subunit Naa10p revealed that the substantial conformational change in the α1-loop-α2 region of Naa10p was driven by interacting with auxiliary subunit Naa15p, for a substrate preference from Glu to Ser^16^.

As compared with eukaryotes, in archaea, knowledge of the N-terminal protein acetylation is relative limited^17^. A BLAST search identified Ard1 from Crenarchaeal *Sulfolobus solfataricus* (SsArd1) as a homolog of the Ard1 subunit of yeast NatA and Naa10p subunit of human NatA. However, no *Sulfolobus* homolog of the auxiliary subunit of NatA was found, so SsArd1 alone may be responsible for the protein acetylation. Limited proteomic survey of N-terminal acetylation in *S. solfataricus* revealed that the acetylated N-terminal sequence began with Ser, Ala and Met-Glu residues at the terminus^18^. One of the proteins to be acetylated is Alba, a major archaeal chromatin protein^19^. Alba possesses a Ser residue at the N-terminus and is the best protein substrate of SsArd1 *in vitro*. The *Sulfolobus* single-stranded DNA-binding protein (SSB) with Met-Glu at the N-terminus is not a traditional substrate for eukaryotic NatA but could be acetylated by SsArd1^18^. Notably, SsArd1 acetylates SSB more quickly than Holliday junction-resolving enzymes Hjc and Hje, which have Ala residues at their N-termini^18^. The sequence specificity may be more relaxed in SsArd1 than eukaryotic proteins.

To elucidate the molecular basis for substrate specificity and the catalytic mechanism of SsArd1, we determined the X-ray crystal structures of SsArd1 complexed with CoA with and without a cognate N-terminal substrate. Structure-guided mutagenesis and accompanying biochemical characterization were used to derive structure function correlations underlying SsArd1 acetylation and substrate selection.

## Results

### Overall structures of SsArd1 complexes

The purified protein was crystallized in complex with CoA and with CoA/peptide. The crystals of the 2 complexes belong to the orthorhombic *P*2_1_2_1_2_1_ space group with similar unit cell parameters, and the crystallographic asymmetric unit of both structures contained one molecule only. The crystal structure of the SsArd1-CoA binary complex was determined at 2.13 Å resolution and the SsArd1–CoA/peptide ternary complex at 1.84 Å. The binary and ternary complex structures were solved by molecular replacement and refined to an *R*_work_/*R*_free_ of 17.2/21.7% and 19.0/24.6%, respectively, with 99.0% and 98.7% residues, respectively, in the favored region of the Ramachandran plot (Table 1).

**Figure 1:**
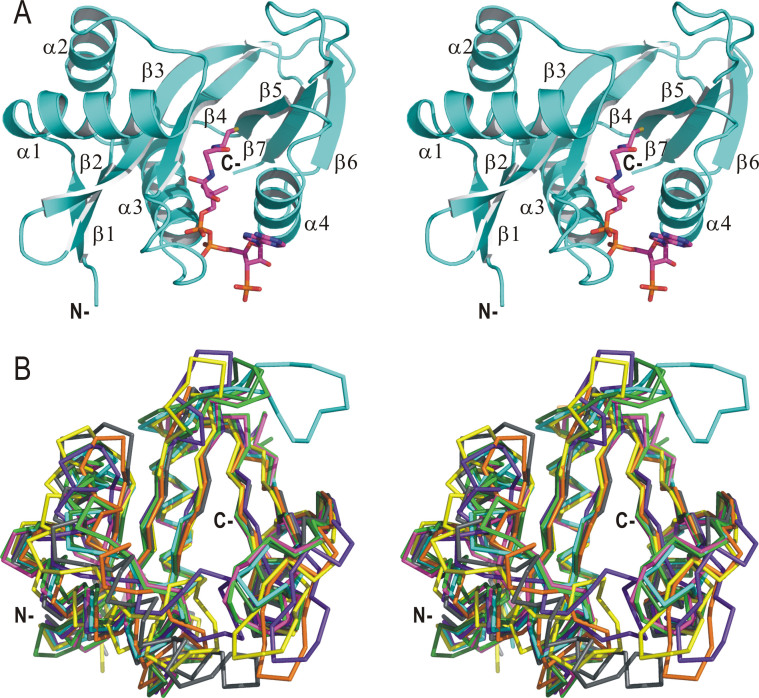
Overall structure and topology of *Sulfolobus solfataricus* Ard1 (SsArd1) complex. (A) Cartoon diagram of the crystal structure of SsArd1 complex with CoA (shown as sticks). Termini and secondary structure elements are labeled. (B) Stereoview of Cα traces representing superimposed crystal structures of SsArd1 (cyan) with similar structures (dark green, 4KVM; magenta, 2PSW; light green, 3TFY; orange, 2CNS; yellow, 2GE3; purple, 3LD2; gray, 3NE7) from the DALI server.

CoA-bound and CoA/peptide-bound SsArd1 structures were almost identical and could be superimposed, with root mean square deviation (RMSD) 0.12 Å for all Cα atoms. SsArd1 has a mixed α/β-fold with 7 β-sheet surrounded by 4 α-helices and contains 4 conserved motifs, a feature unique to the GCN5-related N-acetyltransferase (GNAT) family^20^ (Figure 1A). In the SsArd1 structure, the 2 central parallel β-strands 4 and 5 are located in the middle and cut through the center of the structure to divide the α1 and α2 helices on one side of the central β-sheet and α3 and α4 on the opposite side. The orientation of each close β-strands is antiparallel, except for strands β4 and β5. The sheet features a V-shape appearance of central β-strands 4 and 5 because of the β4 and β5 interaction and is a critical characteristic for CoA binding^21^.

**Figure 2:**
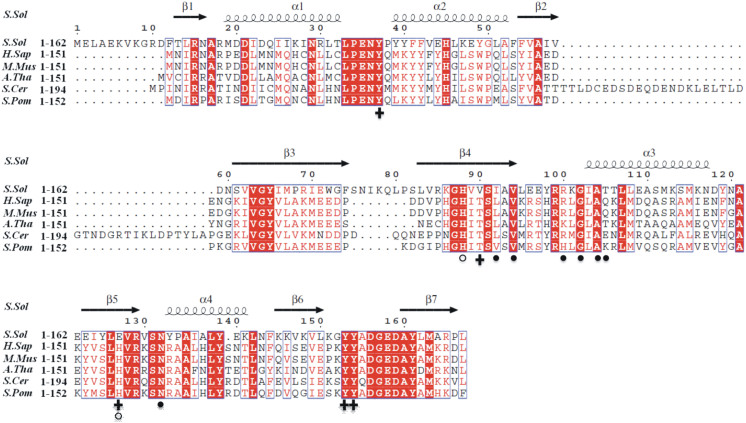
Sequence alignment of SsArd1 with NatA homologs. Multiple sequence alignment of SsArd1 and homologues from *Homo sapiens (H.sap), Mus musculis (M.mus), Arabidopsis thaliana (A.tha), Saccharomyces cerevisiae (S.cer)* and *Schizosaccharomyces pombe (S.pom)*. Sequences were aligned by use of ClustalW2 and the figure was generated by use of ESPript 2.2. Identical and similar residues are labeled by white letters on red backgrounds and red letters, respectively. The residues involved in interacting with CoA and the peptide by hydrogen bonds and catalysis are indicated with a black circle (•), plus signs (+) and white circle (○), respectively.

Structural homology search with the DALI server^22^ revealed that SsArd1 is similar to eukaryotic and prokaryotic Nats in structure (Figure 1B), with RMSD ~2.3 Å (Table 2), despite extremely low amino acid sequence identity (maximal 35%) (Figure 2). The related structures are Naa10p from *Schizosaccharomyces pombe* (4KVM)^16^, MAK3 homolog from *Homo sapiens* (2PSW), Naa50p from *H. sapiens* (3TFY)^23^, RimI form *Salmonella typhimurium* (2CNS)^21^, Probale acetyltransferase from *Agrobacterium tumefaciens* (2GE3), smu.2055 from *Streptococcus mutans* (3LD2), and PaiA from *Thermoplasma acidophilum* (3NE7)^24^. The structure of Nat is conserved from ancestral to prokaryotic and eukaryotic cells and in different types of substrates of recognition.

### Coenzyme A (CoA) binding site on SsArd1

**Figure 3:**
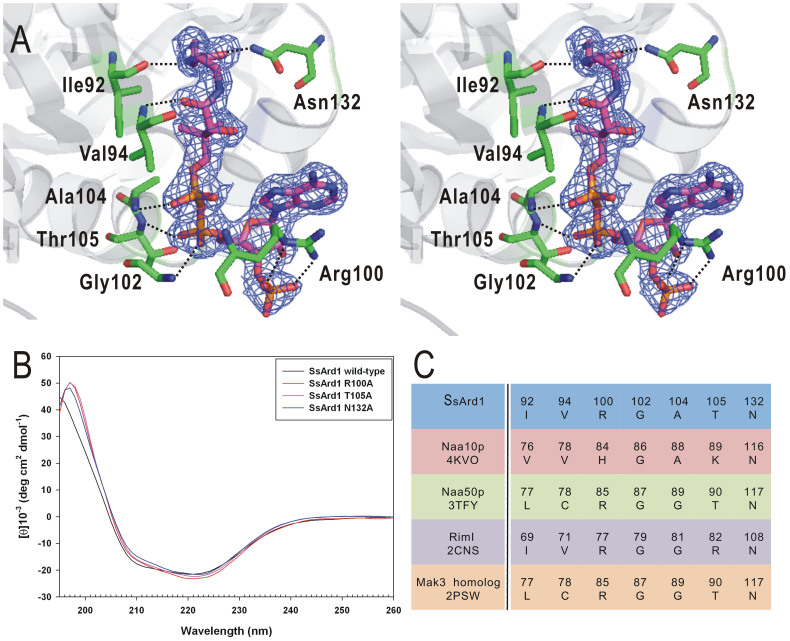
Structural analysis of CoA bound to SsArd1. (A) Stereoview of CoA bound to SsArd1 via a hydrogen bond network involving Ile92, Val94, Arg100, Gly102, Ala104, Thr105 and Asn132. Hydrogen bonds are shown as black dashed lines. A *2F*_o_ - *F*_c_ electron-density map for CoA at counter level of 2.0. (B) Far-UV circular dichroism spectra from 260 to 195 nm for wild-type and various mutated SsArd1. The sample concentration was 30 μM at pH 8.0. (C) Critical residues for CoA binding from SsArd1 and other Nats with known structure.

From the protein sequence, SsArd1 contains a typical {Arg/Gln}-X-X-Gly-X-{Gly/Ala} motif (Arg99-X-X-Gly102-X-Ala104) important for CoA recognition and binding for other members of the GNAT superfamily^25^. In the complex structure, the adenosine 3′-phosphate group of CoA is placed on the outside of the tunnel entrance and exposed to the protein surface. The CoA is bound to SsArd1 in a bent conformation in the pyrophosphate region, for pantetheine fitting to the narrow central cleft and the acetyl group reaching the midpoint of the tunnel. Adenosine 3′-phosphate, pyrophosphate and pantothenic acid of CoA contact SsArd1 via many van der Waals interactions and hydrogen bonds. The main-chains of Ile92, Val94, Arg100, Gly102, Ala104 and Thr105 contribute to hydrogen bonding with CoA. The side-chains of Arg100, Thr105 and Asn132 contact with the 3′-phosphate of adenosyl moiety, pyrophosphate and pantothenic acid, respectively, by hydrogen bonds (Figure 3A).

To elucidate the function of the 3 side-chains of Arg100, Thr105 and Asn132, interacting with CoA, these SsArd1 residues were single-point mutated to Ala for enzyme activity assays. Similar far-UV CD spectra for wild-type SsArd1 and the 3 mutants indicated their identical folds, which were not altered by amino acid replacement (Figure 3B). To determine the kinetic parameters of acetyl-CoA, we used a saturating concentration of Alba peptide as a substrate. With the Michaelis-Menten equation, the *k*_cat_ and *K_m_* values of acetyl-CoA for SsArd1 at 65°C were calculated as 33.57 ± 1.65 min^−1^ and 67.17 ± 7.48 μM, respectively. As compared with wild-type SsArd1, R100A, T105A and N132A mutants showed ~7, ~3 and ~4.5-fold increase in *K_m_*, with no significant difference in *k*_cat_ (Table 3). Therefore, these residues of SsArd1 may be important for CoA binding but not catalytic activity. The crystal structure of SsArd1 in complex with CoA revealed that Arg100 was the only residue contributing two hydrogen bonds to the 3′-phosphate of adenosyl moiety. The side-chain of Arg100 served as a position of adenosine moiety of CoA located at the cave formed by the α4 and β4-α3 loop. The R100A mutant showed reduced binding affinity correlated with loss of hydrogen bonds between SsArd1 and AcCoA. The side-chain of Asn132 for SsArd1 formed a hydrogen bond with the pantothenic acid of CoA. Consistently, the N132A mutant showed slightly lost binding affinity for AcCoA.

**Figure 4:**
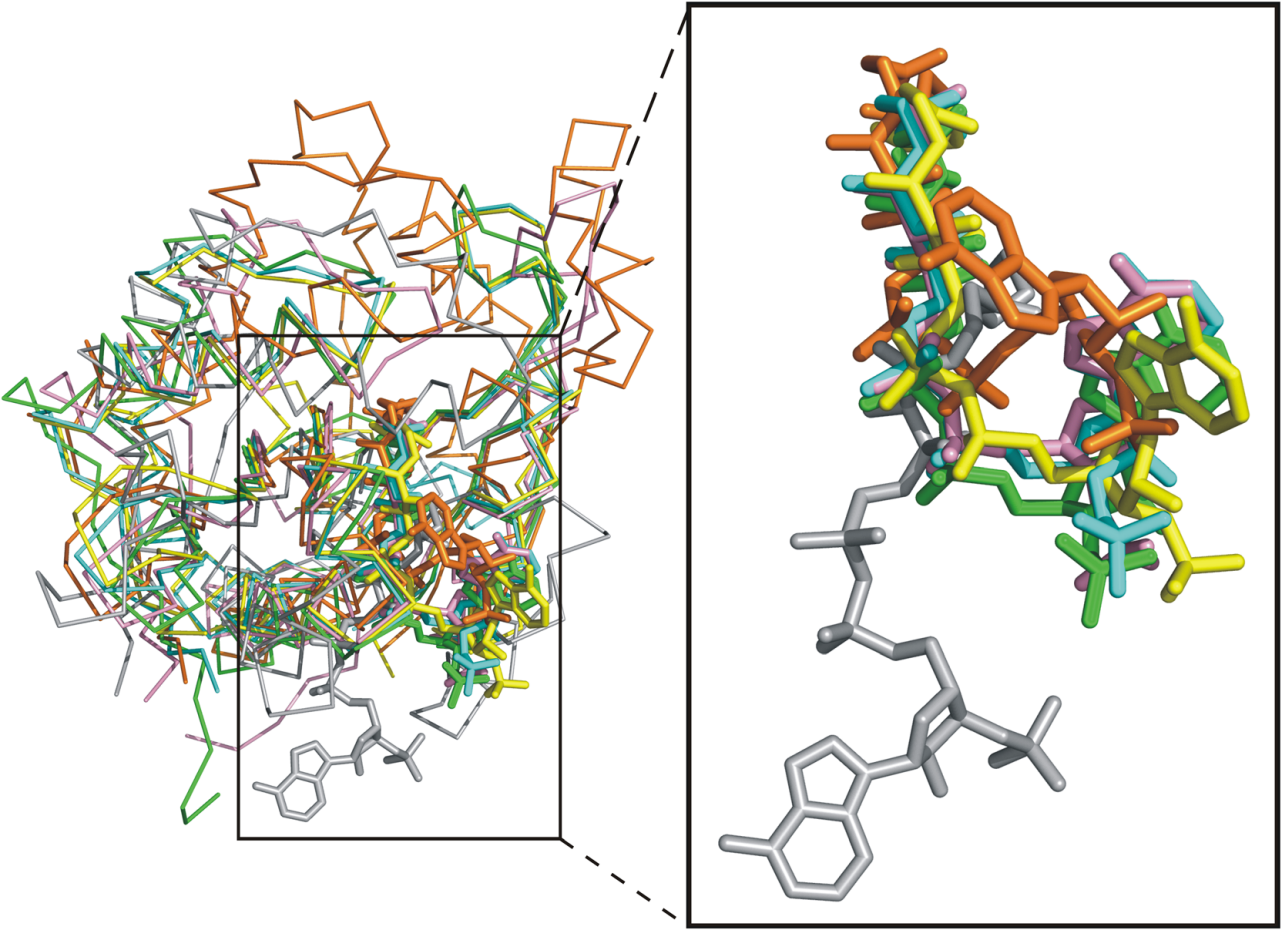
Structural comparison of GNAT with different CoA conformation. Superimposition of SsArd1 (cyan) with *Homo sapiens* Naa50p (green) (RMSD 1.08 Å for 112 Cα atoms), *Salmonella typhimurium* RimI (pink) (RMSD 0.75 Å for 67 Cα atoms), *Schizosaccharomyces pombe* Naa10p (yellow) (RMSD 0.68 Å for 129 Cα atoms), *Homo sapiens* spermidine/spermine acetyltransferase (gray) (RMSD 1.08 Å for 68 Cα atoms), and *Drosophila melanogaster* dopamine N-acetyltransferase (orange) (RMSD 4.00 Å for 74 Cα atoms) (left panel), plus close-up of the acetyl-CoA/CoA from the left panel. The structures and acetyl-CoA/CoA are shown as a ribbon diagram and stick, respectively.

Structural alignment of SsArd1 with Nats with known structure, including Naa10p^16^, Naa50p^23^, RimI^21^ and MAK3 homolog, showed similar orientation and position of CoA-binding residues, Ile92, Val94, Arg100, Gly102, Ala104, Thr105 and Asn132 (Figure 3C), but these amino acids of SsArd1 are not all conserved across homologs of other kingdoms. Arg100 and Asn132 of SsArd1 are highly conserved only within the Nats family. The second residue of the acetyl-CoA binding motif, {Arg/Gln}-X-X-Gly-X-{Gly/Ala}, is a glycine and is highly conserved for GNATs; it acetylates small molecules such as polyamine^26^ and dopamine^27^ but a larger-residue Arg for the Nats protein family. Structural comparison among these GNATs revealed significantly different orientation of adenosine 3′-phosphate groups of CoA and toward the opposite side (Figure 4). The polyamine or dopamine acetyltransferase containing the smaller glycine residue on the second position of the motif allows for a more flexible adenosine 3′-phosphate group of CoA. In contrast, the positive-charged guanidino group of the Arg100 residue of SsArd1 occupied the space and formed a cation-pi interaction with the adenosine group of CoA, which may force the acetyl group less deep into the catalytic pocket for larger protein N-terminal residues.

### Substrate specificity of SsArd1

**Figure 5:**
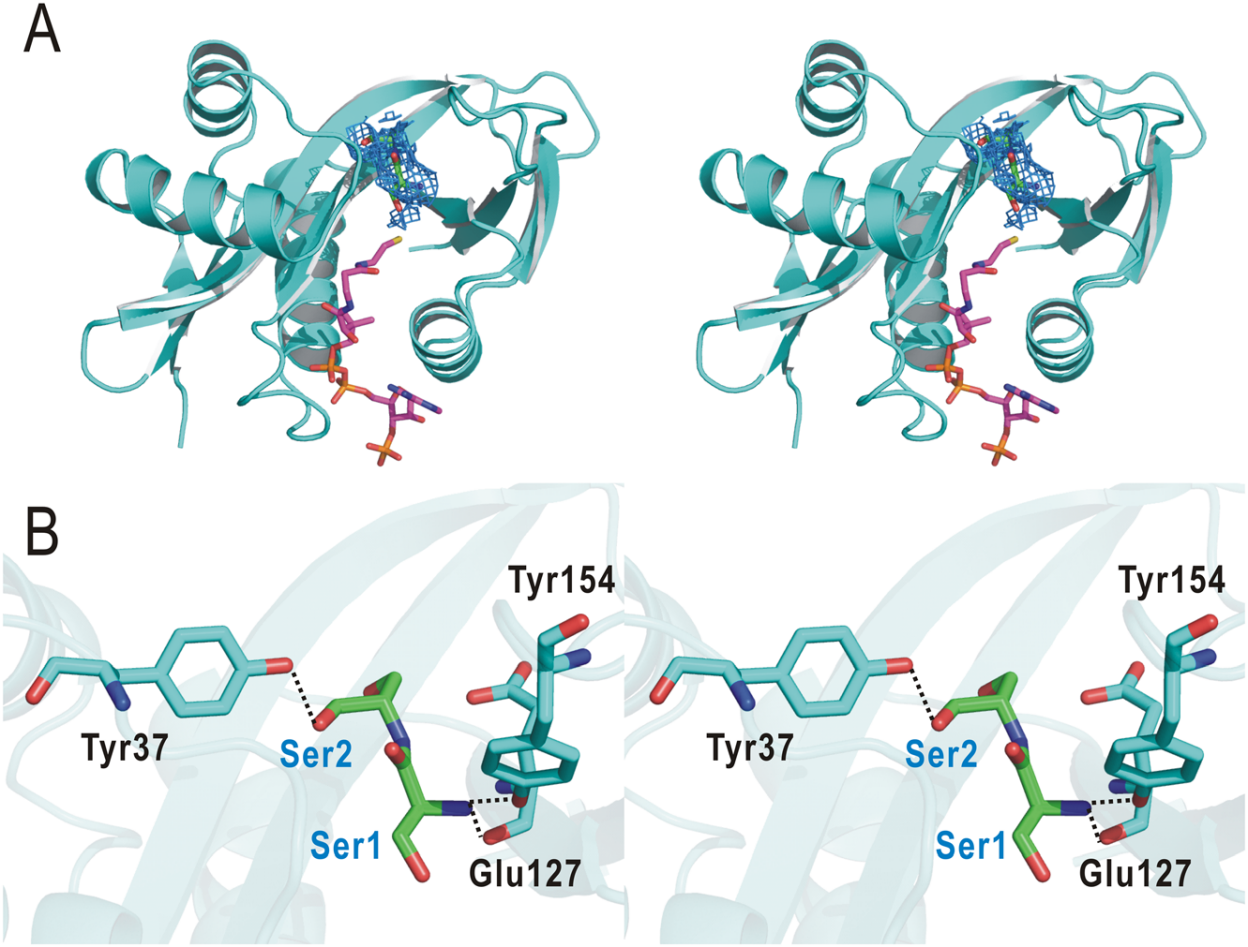
The interaction of SsArd1 with Alba substrate peptide. (A) Stereo representation of the crystal structure of SsArd1 complex with CoA (magenta) and peptide (green). A *F*_o_ - *F*_c_ omit electron density map (prior to modeling the ligand) is contoured at the 2.0 σ level (blue). (B) Stereo diagram of a stick representation of the interaction between the SsArd1 and peptide. Substrates are in green and numbered 1–2 sequentially from the N-terminus. Hydrogen bonds are depicted as black dashed lines.

In the crystal structure of the SsArd1–CoA/peptide complex, we identified a clear electron-dense *F_o_–F_c_* map corresponding to the first 2 residues of the 6-mer Alba peptide. The Alba peptide fragment is located in the pocket formed between the α1-α2 loop, β6-β7 loop, and β4 and β5. The first 2 residues of Alba bound to SsArd1 via a number of van der Waals interactions and hydrogen bonds (Figure 5A). The main-chain of Glu127 and side-chain of Tyr154 interacted with the main-chain of the first residue Ser by a hydrogen bond. In addition, the main-chain carbonyl of the second Ser formed a hydrogen bond to the side-chain of Tyr37 (Figure 5B).

The NatA complex of eukaryotes is substrate-specific for the N-terminal end of the protein sequence with Ser, Thr, Ala, Val, Gly and Cys after the first Met is cleaved by methionine aminopeptidases^10^,^12^. SsArd1 is homologous with the catalytic subunit of eukaryotic NatA complex. Alba possesses a SSGTPT sequence at the N-terminus and represents the highest level of acetylation for SsArd1^18^. In addition, as an SsArd1 substrate, SSB and Hjc from *S. solfataricus* were acetylated with ~16% and ~12% activity, respectively, as compared with that of Alba. Hjc possesses an N-terminal AAKKRG sequence, preferred by NatA; however, SSB contains the N-terminal sequence MEEKVG, preferred by eukaryotic NatE but not NatA^18^.

**Figure 6:**
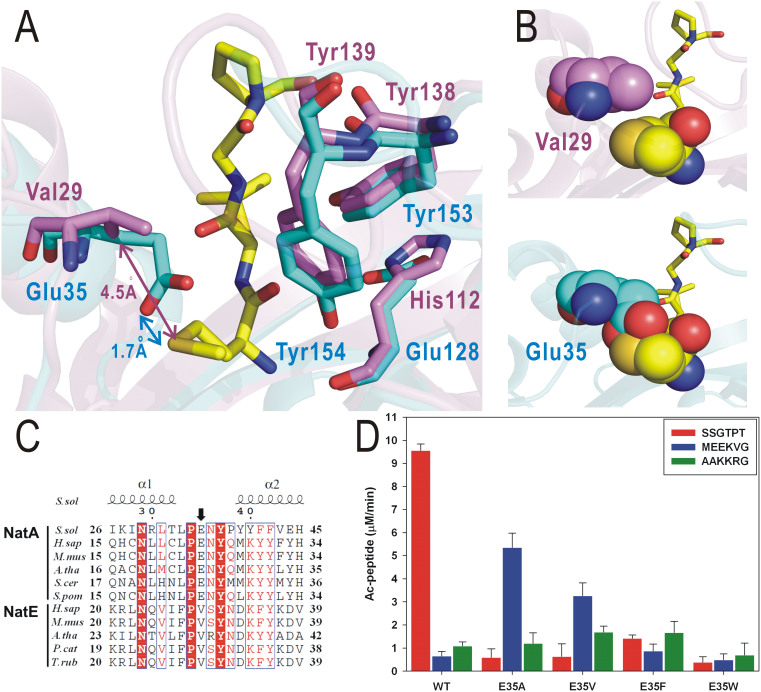
Substrate specificity of SsArd1 (NatA) as compared with hNaa50p (NatE). (A) Structural superimposition of SsArd1 (cyan) with hNaa50p (magenta) in complex with MLGP (Met-Leu-Gly-Pro) substrate (yellow). (B) Spheres represent the interspace between Val29 of hNaa50p (magenta) and the first residue, Met (yellow) of the substrate from hNaa50p (upper panel) and Glu35 of SsArd1 (cyan) and the first residue, Met (yellow) of the substrate from hNaa50p (lower panel). (C) Sequence alignment of NatA and NatE families from *H. sapiens* (*H.sap*), *Mus musculis* (*M.mus*), *Arabidopsis thaliana* (*A.tha*), *Saccharomyces cerevisiae* (*S.cer*), *S. pombe* (*S.pom*), *Physeter catodon* (*P.cat*) and *Takifugu rubripes* (*T.rub*). Sequences were aligned by use of ClustalW2 and the figure was generated by use of ESPript 2.2. Identical and similar residues are labeled by white letters on red backgrounds and red letters, respectively. (D) Enzyme activity assay of wild-type SsArd1 and various single-point Glu35 mutants with different substrate peptides for NatA and NatE.

To clarify the substrate specificity, we analyzed the structure of the human Naa50p-CoA/peptide complex (belonging to NatE, PDB code: 3TFY) with the high resolution SsArd1-CoA/peptide complex. The core fold of SsArd1 is very similar to that of human Naa50p (hNaa50p), despite low sequence identity (24.1%) (Figure 1B). Nevertheless, SsArd1 superimposes well with hNaa50p, with RMSD 1.0 Å for 110 Cα atoms. Although hNaa50p shares the fold core with SsArd1, SsArd1 possesses a different substrate for acetylation. Structural alignment of ternary complexes of SsArd1 and hNaa50p revealed that residues of substrate binding for hNaa50p, Val29, His112, Tyr138 and Tyr139 were located in almost the same orientation and overlapped with the residues Glu35, Glu127, Tyr153 and Tyr154 for SsArd1 (Figure 6A). However, the hydrophobic Val29 side-chain of hNaa50p seemed to be responsible for substrate specificity of protein with N-terminal Met. The distance from the sulfur atom of Met is ~4.5 Å for the side-chain of Val29 in hNaa50p but ~1.7 Å for Glu35 in SsArd1. A comparison of Val29 of hNaa50p and Glu35 of SsArd1 in these ternary complexes showed that the side-chain of Glu35 from SsArd1 is too close and features steric clashes against the side-chain of the first Met residue of the substrate for hNaa50p (Figure 6B). In contrast, there is no space for the substrate of hNaa50p to accommodate the pocket in SsArd1. Residue Glu35 of SsArd1 may play a critical role for substrate specificity in Nα-acetylation.

Sequence alignment revealed that the Glu and Val residues are highly conserved in NatA and NatE, respectively (Figure 6C). To investigate whether the size influenced the substrate specificity of Nα-acetylation, we substituted smaller and larger hydrophobic side-chain residues, Ala, Phe, and Trp, as well as Val, the conserved residue from NatE with Glu35 of SsArd1.

Enzyme activity assays were performed with 3 different substrate peptides. For the Alba substrate peptide, mutants with Glu35 of SsArd1 replacing Ala, Val, Phe and Trp all showed reduced activities to <10% as compared with wild-type SsArd1 (Figure 6D). Notably, substituting Glu35 for Ala and Val of SsArd1 resulted in approximately 8- and 5-fold increased activity, respectively, as compared with the wild type for SSB substrate. These results may explain the structural basis of substrate preferences and differences in NatA and NatE; Glu35 may have a critical role in restricting the substrate specificity to smaller residues such as Ser at the N-terminal end. E35A and E35V mutants alter the N-terminal substrate specificity and allow the larger N-terminal end residue of the substrate to be accommodated in a substrate-binding pocket. For the Hjc peptide substrate with N-terminal Ala, wild-type SsArd1 reserved only ~10% enzyme activity as compared with the Alba substrate with Ser. However, E35V and E35F mutants showed an increase of 2-fold enzyme activity for the Hjc substrate peptide as compared with the wild-type SsArd1 and E35A mutant. Alanine, the first residue of the Hjc substrate, may make room for bulky Val and Phe residues, along with the contribution of the hydrophobic interaction. However, the Trp-substituted SsArd1 mutant showed very low enzyme activity with all 3 substrate peptides because the binding pocket was occupied by the huge side-chain of tryptophan. Thus, the size and properties of residue corresponding with Glu35 of SsArd1 may play an important role for substrate specificity in SsArd1.

## Discussion

**Figure 7:**
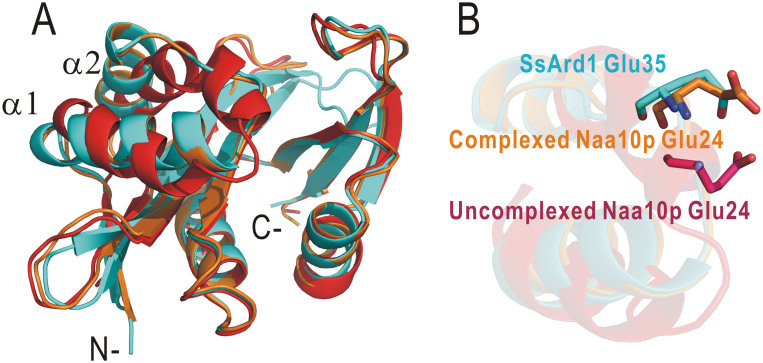
Structural comparison of SsArd1 with eukaryotic NatA catalytic subunit Naa10p. (A) Structural superimposition of SsArd1 (cyan) with complexed Naa10p (orange) and uncomplexed Naa10p (red). (B) The zoom-in view of the key residue for substrate specificity is from the structural alignment represented in A.

Protein Nα-acetylation is a ubiquitous post-translational modification present from archaea to mammalian cells and is involved in a number of biological functions including as a signal for protein degradation, an inhibitor of endoplasmic reticulum translocation and a mediator of protein complex formation in eukaryotes^8^,^28^,^29^. The precise substrate recognition for Nats remains poorly understood. The catalytic subunit Naa10p of NatA is conserved from archaea to higher eukaryotes. A BLAST search and sequence analysis revealed that SsArd1 from thermophilic archaea belongs to the NatA family and acetylates the substrate N-terminal sequence including Ser, Ala and Thr. Interestingly, this N-acetyltransferase (Nat) orthologs in *Sulfolobus solfataricus* as a potential Nat catalytic subunit, but no obvious counterpart to a regulatory partner. The result suggests that the mechanism of substrate selection within this branch of the Nat family may be different from that observed in eukaryotes. We used SsArd1 to understand the substrate specificity and recognition mode of NatA. The crystal structure of SsArd1 was similar for both complexed and uncomplexed forms of the catalytic subunit Naa10p. However, the α1-loop-α2 segment of SsArd1 almost overlapped with the corresponding region of Naa10p in the complexed but not uncomplexed form (Figure 7A). The substantial conformational change of the α1-loop-α2 in Naa10p from the uncomplexed to complexed form leads to the side-chain of Glu24 on the loop repositioned by ~4 Å to facilitate the coupling of substrate binding specificity from Glu to Ser^16^. Superimposition of SsArd1 with both forms of Naa10p revealed that residue Glu35 of SsArd1 located almost in the same position as Glu24 of the complexed Naa10p (Figure 7B). SsArd1 may be an ancestral monomeric NatA with a constricted protein substrate-binding pocket. Eukaryotic NatA may exist as heterodimer with a homolog of SsArd1 and have an additional unique auxiliary subunit, which facilitates the complicated modulation of substrate specificity for eukaryotic protein acetylation.

We demonstrated that Glu35 of SsArd1, the residue conserved in the NatA family, has a critical role for the unique substrate specificity. SsArd1 has a broader sequence specificity for substrate as compared with eukaryotic Nats. Although SsArd1 has a little enzyme activity with NatE, the major substrate acetylated by SsArd1 is Ser more than Met-Glu. Superimposition of SsArd1 with Naa50p (NatE) revealed that the side-chain of the residue Glu35 would clash with the Met residue of the substrate peptide, so Glu35 of SsArd1 may be important for excluding these N-terminal substrates. This suggestion is consistent with the importance of the corresponding Val29 residue of human Naa50p for N-terminal Met recognition and acetylation^23^. Interestingly, E35A and E35V mutants of SsArd1 altered the substrate preference and catalyzed the N-terminal end of the substrate with the NatE substrate Met-Glu instead of Ser. Glu35 of SsArd1 and the corresponding residue of NatA replaced by the smaller or more hydrophobic residue in the binding pocket may be able to accommodate an N-terminal Met-Glu containing the substrate peptide and play an equivalent role in catalysis. The different activity for this substrate-mutant combination further supports the importance of Glu35 in the SsArd1 catalytic mechanism.

This study gives the first molecular details for substrate recognition for the N-terminal Ser substrate in the NatA family and sheds light on the molecular mechanism of substrate binding and specificity in the family.

## Methods

### Protein expression and purification

DNA encoding the wild-type and various mutants of SsArd1 was amplified by PCR and subcloned into a pET28a vector (Novagen) for fusing the gene to a C-terminal His_6_-tag. The final constructs were verified by DNA sequencing. All of the recombinant plasmids were transformed into *Escherichia coli* strain BL21 (DE3). Cells were grown in lysogeny broth (LB) at 37°C until OD_600_ 0.6, and protein overexpression was induced for 4 h by the addition of 1.0 mM isopropyl 1-thio-β-D-galactopyranoside. The cultures were harvested at 6,000 rpm for 30 min. Cell pellets were resuspended in buffer A (20 mM Tris-HCl, pH 7.5, 100 mM NaCl, 1 mM DTT), then lysed by sonication on ice, and insoluble pellets were removed by centrifugation at 17,418 *g* for 30 min at 4°C. The supernatant was filtered through a 0.45-μm filter membrane to remove cell debris before being applied to Ni-Sepharose columns. Columns were washed with buffer B (buffer A containing 50 mM imidazole) and target protein was eluted with buffer C (buffer A containing 300 mM imidazole). Further purification involved size-exclusion chromatography with a HiPrep 16/60 Sephacryl S-200 HR column (GE Healthcare) with buffer D (20 mM Tris-HCl, pH 7.5, 200 mM NaCl, 1 mM DTT). Peak fractions were examined by Coomassie blue-staining SDS-PAGE. Fractions containing the target protein were pooled and concentrated to 6 mg/ml.

### Crystallization and data collection

The preliminary crystallization screening for SsArd1 with CoA involved a sitting-drop vapour-diffusion method at 283 K with the commercial kits Crystal Screen, Crystal Screen 2, PEGRx1, PEGRx2, PEG/Ion, PEG/Ion 2, SaltRx1, SaltRx2, Natrix and Index (Hampton Research); MD1-13 (Molecular Dimensions); and Wizard I and Wizard II (Emerald BioSystems) in a 96-well plate. Crystals of the SsArd1-CoA complex were obtained with 30% w/v PEG 4,000, 0.1 M sodium acetate trihydrate, pH 4.6, and 0.2 M (NH_4_)_2_SO_4_. To improve crystals of quality and size for diffraction, the crystallization conditions were optimized by varying the pH, precipitant and salt concentration. Crystal optimization in 24-well plates involved mixing 1 μL protein with 1 μL reservoir against 300 μL reservoir at 283 K. The highest-diffraction quality data of SsArd1 crystal was collected from the optimal condition 5.0% w/v PEG 4,000, 0.1 M sodium acetate trihydrate pH 4.2, and 0.1 M (NH_4_)_2_SO_4_. To obtain the ternary SsArd1 complex, SsArd1 crystals were further soaked with 2 mM Alba peptide (SSGTPT) and dehydrated over 12.5% w/v PEG 4,000. X-ray diffraction datasets were collected by SPXF beamline BL13B1 and BL15A1 at the National Synchrotron Radiation Research Center (NSRRC, Hsinchu, Taiwan) with ADSC Q351r (BL13B1) and Rayonix MX300HE (BL15A1) charge-coupled device detectors, respectively. The diffraction data were processed and scaled by use of HKL-2000^30^.

### Structure determination and refinement

The crystal structure of SsArd1 was determined by a molecular replacement method with use of BALBES^31^, by using the apo-form structure of SsArd1 (PDB code: 2X7B) as a search model. Crystallographic refinement involved repeated cycles of conjugate-gradient energy minimization and temperature-factor refinement performed with REFMAC5^32^ and PHENIX^33^. Amino-acid side chains and water molecules were fitted into 2*F*_o_-*F*_c_ and *F*_o_-*F*_c_ electron-density maps by using Coot^34^. The models were evaluated by use of PROCHECK^35^ and MOLPROBITY^36^. The final coordinates and structure factors of binary and ternary SsArd1 complexes were deposited in the Protein Data Bank under accession codes 4R3K and 4R3L, respectively. The data collection and structure refinement statistics are in Table 1.

### Acetyltransferase activity assay

The 50-μL reaction mixture contained 2 μM enzyme solution (20 mM Tris-HCl, pH 8.0, 100 mM NaCl, 1 mM EDTA), substrate solution (20 mM Tris-HCl, pH 8.0, 100 mM NaCl, 1 mM EDTA and variable concentrations of peptide substrate) and 1 mM acetyl-CoA (20 mM Tris-HCl, pH 8.0, 100 mM NaCl, 1 mM EDTA). The reaction mixture was incubated at 65°C for 10 min and stopped by adding 50 μL stop solution (20 mM Tris-HCl, pH 8.0, 100 mM NaCl, 6 M Urea). Then, 100 μL Ellman's reagent (20 mM Tris-HCl, pH 8.0, 100 mM NaCl, 1 mM EDTA and 2 mM DTNB) was added for incubation at 25°C for 10 min. The absorbance values were recorded at wavelength 412 nm on a 96-well plate. The 50-μL standard concentration of CoA (20 μM to 400 μM) was reacted with 50 μL stop solution and 100 μL Ellman's reagent. The raw data were fitted to a Michaelis-Menten equation in SigmaPlot to obtain the steady-state kinetic parameters.

Three N-terminal 6-mer peptides for enzyme assays were synthesized and named Alba (SSGTPT), SSB (MEEKVG), and Hjc (AAKKRG), derived from native substrate proteins Alba, SSB, and Hjc, respectively. The peptides were verified by MALDI-TOF MS (GenScript Co., USA).

### Circular dichroism (CD) spectroscopy

CD experiments involved a Jasco J-810 spectropolarimeter (Jasco International, USA) with a Peltier effect temperature controller (Jasco PTC-423S). Secondary structure determination spectra of wild-type and mutated SsArd1 were examined with 30 μM protein in 20 mM phosphate buffer, pH 8.0, and 2 mM NaCl at 25°C. The spectra were measured in a 1-mm quartz cuvette at wavelength 195 to 260 nm. All samples were centrifuged at 10,000 g for 10 min before analysis. CD spectra for the SsArd1 structure at different temperatures were obtained by heating the sample from 25 to 95°C. The data collection parameters were scanned rate 50 nm/min, response time 1 s, sensitivity 100 mdeg, accumulation 3, heating rate 1°C/min and delay time for collection 60 s. The reversibility of the temperature effect was checked by cooling the sample to 25°C with the same parameters. Baseline subtraction, smoothing and data normalization involved use of SigmaPlot. The CD data are shown as mean residue ellipticity units (deg cm^2^ dmol^−1^).

## Additional information

**Accession codes:** The atomic coordinates and structure factors (codes 4R3K for SsArd1-CoA and 4R3L for SsArd1-CoA/peptide) described in the present paper have been deposited in the Protein Data Bank (http://www.pdb.org/)

## Supplementary Material

Supplementary Information Supplementary Information

## Figures and Tables

**Table 1:** X-ray crystallographic data collection and refinement statistics for SsArd1

Crystal parameters		
Crystal	SsArd1-CoA complex	SsArd1-CoA-peptide complex
Space Group	*P*2_1_2_1_2_1_	*P*2_1_2_1_2_1_
Unit Cell Parameters		
a, b, c (Å)	35.4; 58.2; 85.3	35.2; 58.0; 84.1
α,β,γ (°)	90, 90, 90	90, 90, 90
Monomers per		
Asymmetric Unit Cell	1	1

Data collection		
Wavelength (Å)	1.00	1.00
Resolution Range (Å)	30.0–2.13 (2.21–2.13)	30.00–1.84 (1.90–1.84)
Unique Reflections	10196	15532
I/σ	13.2 (4.06)	16.5 (3.47)
*R*_merge_a,b (%)	7.8 (48.2)	4.4 (48.3)
Completeness^a^ (%)	99.6 (99.5)	99.8 (99.7)
Redundancy^a^	5.6 (5.6)	4.7 (4.8)

Refinement statistics		
Resolution (Å)	2.13	1.84
*R*_work_ (%)/*R*_free_ (%)^c^	17.2/21.7	19.0/24.6
R.M.S.D.		
Bonds (Å)	0.007	0.011
Angles (°)	1.12	1.18
No. atoms		
Protein	1292	1292
CoA	48	48
Peptide	-	12
Water	39	85
Mean B-factor (Å^2^)		
Protein	42.7	29.6
CoA	55.3	35.8
Peptide	-	78.4
Water	46.8	40.4
Ramachandran plot (%)		
Favored	99.0	98.7
Allowed	1.0	1.3
Outliers	0.0	0.0

aValues in parentheses are for the highest resolution shell.

b*R*_merge_ = Σ_h_Σ_i_|*I_h_,_i_-I_h_*|/Σ_h_Σ_i_*I_h_*,*_i_*, where *I_h_* is the mean intensity of the *i* observations of symmetry related reflections of *h*.

c*R*_work_/*R*_free_ = Σ|*F_obs_*-*F_calc_*|/Σ*F_obs_*, where *F_calc_* is the calculated protein structure factor from the atomic model (*R*_free_ was calculated with 5% of the reflections selected).

**Table 2:** Structural comparisons of SsArd1 with structurally similar proteins using DALI server

PDB code	Protein	Z-score	RMSD	%ID
4KVM	N-terminal acetyltransferase A complex catalytic subunit	25.6	1.2	35
2PSW	Human MAK3 homolog	21.6	1.7	26
2OB0	Human MAK3 homolog	21.5	1.7	26
3TFY	N-terminal acetyltransferase 50	21.4	1.7	26
2CNS	N-terminal acetylation of ribosomal S18	21.2	1.8	25
2GE3	Probable acetyltransferase	19.6	2.3	22
3LD2	Putative acetyltransferase	19.1	2.2	23
3NE7	PaiA N-acetyltransferase	18.7	1.5	21

**Table 3:** Kinetic parameters for acetyl-CoA binding for wild-type SsArd1 and its mutants

Enzyme	*K_m_*_, AcCoA_	*k*_cat, AcCoA_	*k*_cat_/*K_m_*
	μM	min^−1^	min^−1^ μM^−1^
Wild type	67.17 ± 7.48	33.57 ± 1.65	5.02×10^−1^ ± 0.31
R100A	466.98 ± 50.83	28.70 ± 2.69	6.15×10^−2^ ± 0.09
T105A	195.89 ± 12.27	34.91 ± 1.23	1.78×10^−1^ ± 0.04
R132A	279.37 ± 75.86	33.36 ± 3.88	1.23×10^−1^ ± 0.21
