# Supplementary material for: Structural Basis for Substrate-specific Acetylation of Nα-acetyltransferase Ard1 from Sulfolobus solfataricus
Source: Sci Rep. 2015 Mar 2;5:8673. doi: 10.1038/srep08673 (PMC5390088; doi:10.1038/srep08673)
Supplement: Supplementary Information [file srep08673-s1.pdf]

Supplementary Information

Structural Basis for Substrate-specific Acetylation of α-acetyltransferase Ard1 from *Sulfolobus solfataricus*

Yu-Yung Chang[1], and Chun-Hua Hsu[1,2*]

[1]Department of Agricultural Chemistry, National Taiwan University, Taipei 10617, Taiwan

[2]Center for Systems Biology; Genome and Systems Biology Degree Program, National Taiwan University, Taipei 10617, Taiwan

[*]To whom correspondence should be addressed:

Chun-Hua Hsu, Department of Agricultural Chemistry, National Taiwan University, Taipei 10617, Taiwan, Tel.: (886) 2-3366-4468; Fax: (886) 2-3366-4468; E-mail: andyhsu@ntu.edu.tw

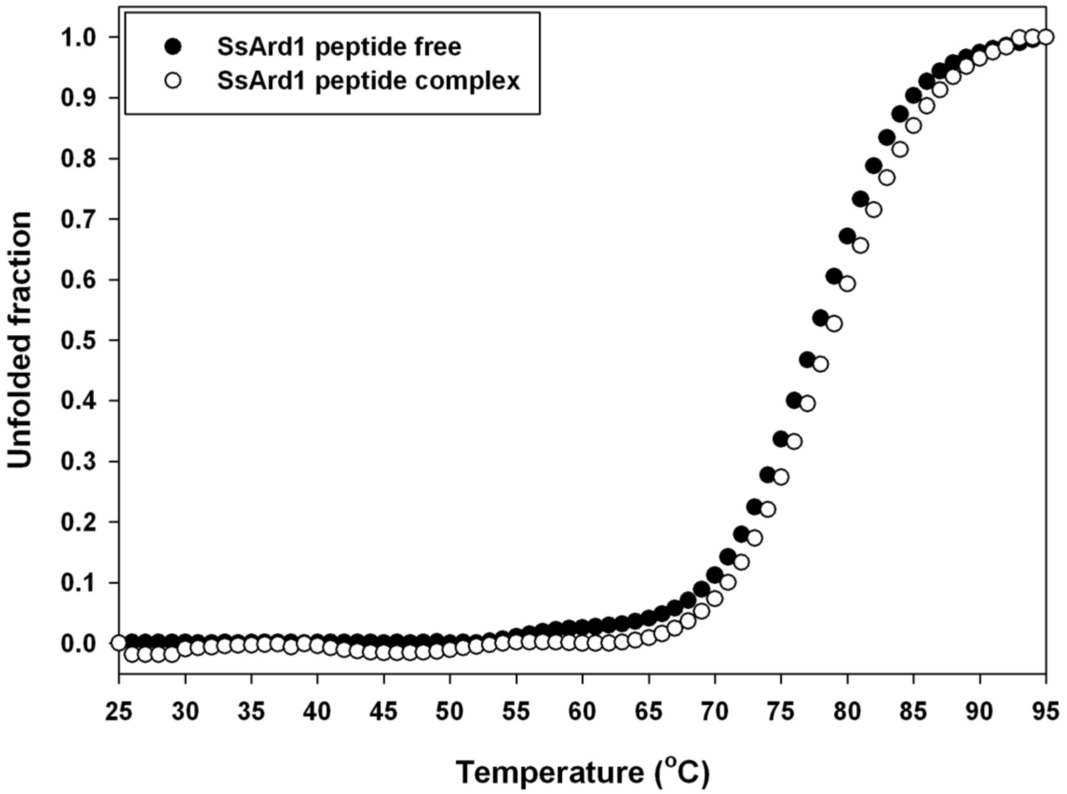

Figure S1. Thermal stabilities of peptide-free and peptide-bound *Sulfolobus solfataricus* Ard1 (SsArd1). The thermal unfolding was monitored by recording the circular dichroism signal at 220 nm in a scanning from 25 to 95°C, at a rate of 1°C/min.

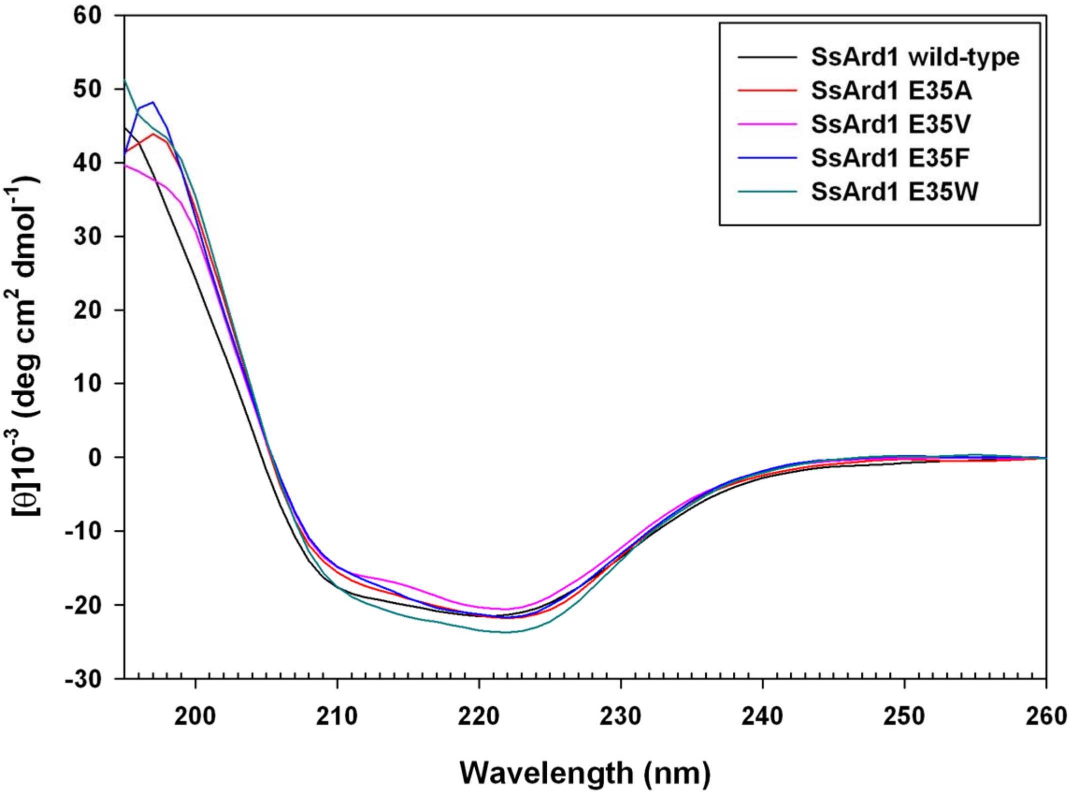

Figure S2. Far-UV circular dichroism spectra from 260 nm to 195 nm for wild-type SsArd1 and its Glu^{35} mutants.

Table S1 Residues of AcCoA binding motif of SsArd1 compare with that of known GNAT structures.

SsArd1	R100	R101	K102	G103	I104	A105
3TFY	R84	R85	L86	G87	I88	G89
2CNS	Q76	R77	R78	G79	L80	G81
4KVO	R83	H84	L85	G86	L87	A88
2FXF	R101	G102	F103	G104	I105	G106
3BJ8	R101	G102	F103	G104	I105	G106
3TE4	R153	G154	L155	G156	I157	A158
